# Impact of an Abdominal Compression Bandage on the Completion of Colonoscopy for Obese Adults: A Prospective Randomized Controlled Trial

**DOI:** 10.1155/2022/6010367

**Published:** 2022-09-06

**Authors:** Ting-Ting Liu, Yi-Teng Meng, Feng Xiong, Cheng Wei, Su Luo, Sheng-Gang Zhan, Yang Song, Ying-Xue Li, Rui-Yue Shi, Jun Yao, Li-Sheng Wang, De-Feng Li

**Affiliations:** Department of Gastroenterology, Shenzhen People's Hospital, The Second Clinical Medical College, Jinan University, The First Affiliated Hospital, Southern University of Science and Technology, Shenzhen 518020, Guangdong, China

## Abstract

**Methods:**

Eligible patients were randomly allocated into the abdominal bandage and conventional groups during a routine colonoscopy. The primary outcome was CCR.

**Results:**

A total of 250 eligible patients were randomly assigned to the abdominal bandage and conventional groups from January 2021 to April 2021. Eleven patients (five in the abdominal bandage group and six in the conventional group) were excluded due to schedule cancellation after randomization, and 239 patients were eventually included in the final analysis. There were no significant differences between the two groups regarding baseline characteristics (*P* > 0.05). Furthermore, no significant differences were observed in terms of advanced adenoma detection rate (AADR), polyp detection rate (PDR), bowel preparation scale (BBPS), bubble scale (BS), and withdrawal time between the two groups (*P* > 0.05). However, compared with the conventional group, the cecal insertion time (CIT) of the abdominal bandage group was significantly shortened (279.00 (234.50–305.75) vs. 421.00 (327.00–485.00), *P* < 0.001), and the CCR (96.7% vs. 88.2%, *P* = 0.01) and adenoma detection rate (ADR) (47.5% vs. 32.8%, *P* < 0.001) were improved. Besides, logistic regression analysis showed that body mass index (BMI) and abdominal compression bandage were associated with CCR.

**Conclusions:**

Abdominal compression bandages could effectively shorten CIT and improve CCR and ADR for obese patients during a routine colonoscopy. This trial is registered with the Chinese Clinical Trial Registry (No. ChiCTR2100043556).

## 1. Introduction

Colorectal cancer (CRC) is the third most commonly diagnosed cancer and the second leading cause of cancer-related death in the world [1]. According to epidemiological evidence, the incidence of CRC has steadily increased in developing and developed countries over the last decades [1–3].

It has been reported that obesity is one of the most predisposing factors associated with CRC. For instance, Soltani et al. [4] have revealed that obesity (body mass index (BMI) ≥ 25 kg/m^2^) is associated with a significantly higher incidence rate of colorectal adenomas compared with normal ones [4]. Kim et al. [5] have found that abdominal obesity is associated with an increased risk of advanced CRC, especially in males, and waist circumference (WC) is an independent risk factor for advanced CRC [5]. In addition, there is a positive association between obesity and an increased risk of colorectal adenomas, CRC, and adenoma recurrence after polypectomy [6–8]. Therefore, more attention should be paid to obese patients to prevent CRC.

Colonoscopy can not only detect early-stage and asymptomatic CRC, but it can also remove the colorectal adenomas, interrupting the adenoma-carcinoma sequence [9–11]. A complete colonoscopy is defined as the insertion of a colonoscope from the anal canal to the cecum, which is critical for reducing the incidence and mortality of CRC. The total colonoscopy rate (CCR) is recommended to be 90% for all colonoscopies and 95% for screening colonoscopies [12]. However, incomplete colonoscopies, termed difficult colonoscopies, may range from 5% to 20% [13, 14]. Additionally, obesity is a critical factor contributing to incomplete colonoscopies because it is challenging to minimize looping in an obese abdomen [15]. However, abdominal compression is a useful ancillary maneuver in colonoscopy, which can help reduce the loop [15]. A few studies have demonstrated that abdominal compression helps decrease patients' pain and improves the CCR during routine colonoscopy [16–18]. However, the efficacy of abdominal compression for obesity remains unclear. Therefore, we aimed to evaluate the impact of an abdominal compression bandage on obese patients during a routine colonoscopy.

## 2. Methods

### 2.1. Study Design

A single-center prospective randomized trial was performed at Shenzhen People's Hospital from January 2021 to April 2021. The study protocol was reviewed by the Human Ethics Committee of Shenzhen People's Hospital and was conducted following the guidelines of the Declaration of Helsinki. Written informed consent was obtained from all participating patients before their enrolment. This study was registered at the Chinese Clinical Trial Registry (No. ChiCTR2100043556).

### 2.2. Study Population

Consecutive adult patients aged 18–80 years were enrolled in Shenzhen People's Hospital by poster advertisement and scheduled for an outpatient colonoscopy. The following criteria were used to identify eligible participants:

The inclusion criteria were set as follows: BMI was calculated as body weight divided by body height squared (kg/m^2^). According to World Health Organization (WHO) criteria, patients with a BMI ≥ 25 kg/m^2^ and a high waist circumference (WC) (>102 cm for males, >88 cm for females) were eligible.

The exclusion criteria were set as follows: abdominal surgery, colonic obstruction or perforation, history of an adenomatous polyp or CRC, Peutz-Jeghers syndrome, familial adenomatous polyposis (FAP), pregnancy, severe cardiac, renal, and respiratory disease, and inflammatory bowel disease (IBD).

### 2.3. Randomization and Blinding

Eligible patients were randomly allocated to either the abdominal bandage group or the conventional group using a computer-generated random number list. The endoscopists were not involved in recruitment and group allocation and were not allowed to enquire about the preparation kits for the patients.

### 2.4. Bowel Preparation

Bowel preparation was administered as described earlier [19]. All patients received a split-dose of 2 L PEG (Shenzhen Wanhe Pharmaceutical Co. Ltd, Shenzhen, China) supplemented with 200 mg simethicone (SIM) (Berlin-Chemie AG, Berlin, Germany).

### 2.5. Colonoscopy Procedure

**Figure 1:**
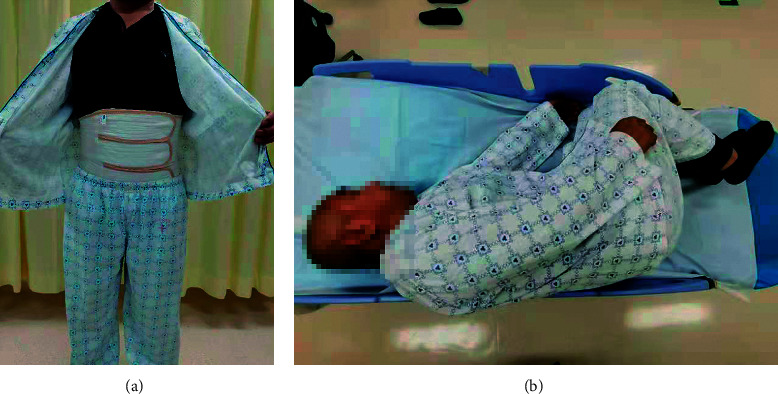
The use of the abdominal bandage.

All colonoscopies were performed by three experienced endoscopists (at least 1,000 colonoscopies per year) using lower gastrointestinal colonoscopes (CF-HQ190L/PCF-H190L, Olympus, Tokyo, Japan) with carbon dioxide insufflation. An abdominal bandage allowing proper abdominal compression was provided by an independent researcher before the colonoscopy in the abdominal bandage group (Figures 1(a) and 1(b)). Ancillary maneuvers, such as position change, were allowed, while abdominal compression using hands was forbidden in the conventional group. All patients received the anesthesia through an intravenous injection of 5 mg midazolam and 50 mg pethidine. All patients underwent colonoscopy between 8 a.m. and 12 p.m.

### 2.6. Data Collection

The endoscopists recorded the information about cecal insertion time (CIT), withdrawal time, bowel preparation quality [Boston Bowel Preparation Scale (BBPS) (BBPS) and bubble scale (BS)], polyp characteristics, and adverse events.

### 2.7. Primary Outcomes

The CCR was the primary outcome. CCR was defined as the proportion of successful cecal intubation.

### 2.8. Secondary Outcomes

Secondary outcomes included CIT, adenoma detection rate (ADR), advanced adenoma detection rate (AADR), polyp detection rate (PDR), adenocarcinoma, adverse events, and withdrawal time, which were defined as previously described [19]. In addition, the BBPS scores and BS were used to assess bowel preparation quality. BBPS was scored on a scale of 0–3 (0, colon segment mucosa not visible: (1) a portion of the colonic mucosa was visible; (2) a minor amount of residual stool covered some segments of the colonic mucosa; and (3) colonic mucosa was adequately visible in all segments). BS was scored based on the amount of foam and bubbles covering the colonic mucosa. The scores ranged from 0–3 ((0) bubbles filled the entire lumen; (1) bubbles filled 25%–50% of the luminal diameter; (2) bubbles filled 5%–25% of the luminal diameter; and (3) no or minimal bubbles).

### 2.9. Sample Size Calculation

To the best of our knowledge, no study has compared these two groups with CCR as the primary outcome measure. Therefore, the sample size was calculated based on a preclinical trial conducted at our hospital with 50 patients each in the abdominal bandage group and the conventional group during the routine colonoscopy procedure. The CCR was 92% and 89% in the abdominal bandage group and conventional group, respectively. Therefore, a sample size of 119 was determined according to an alpha of 0.05, a power of 10%, and a dropout rate of 15% through an online sample size calculator (https://www.cnstat.org/samplesize).

### 2.10. Statistical Analysis

Categorical variables were reported as counts and frequencies (%), and continuous variables were summarized as mean ± standard deviation (SD) or median (interquartile range (IQR)) based on the distribution. For categorical variables, the chi-squared test, Fisher's exact test, and Bonferroni method *χ*2 test were used, while for continuous variables, the Student's *t*-test or Mann–Whitney *U* test was used. In addition, logistic regression was performed to explore the potential factors associated with CCR. All reported *P* values were two-sided, and *P* < 0.05 was considered statistically significant. All analyses were performed using the SPSS 23.0 software package (SPSS Company, Chicago, IL, USA).

## 3. Results

### 3.1. Baseline Characteristics

**Figure 2:**
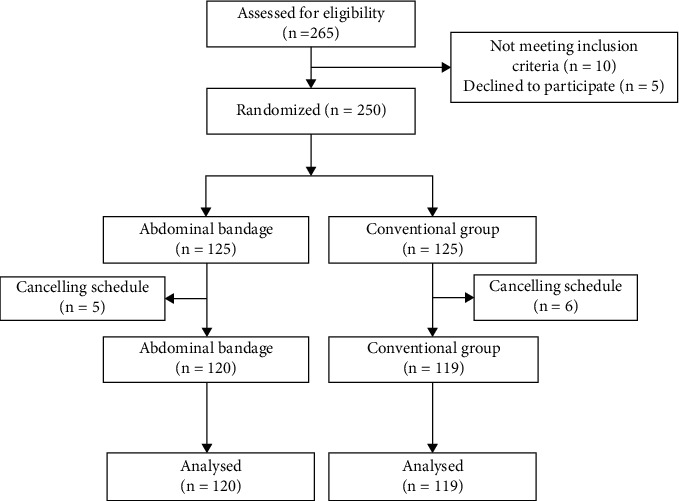
Flow diagram.

Figure 2 shows that 265 consecutive patients were considered for inclusion in the trial, and 250 patients met all the eligibility criteria and were randomly assigned to the abdominal bandage and conventional groups. Of these patients, 11 patients (five patients in the abdominal bandage and six in the conventional group) were excluded because of schedule cancellation. Therefore, 239 patients were eventually included in the final analysis. The baseline characteristics were comparable in terms of gender, age, BMI, WC, previous medical history, and indications for colonoscopy between both groups (*P* = 0.51, *P* = 0.67, *P* = 0.32, *P* = 0.40, *P* = 0.99, and *P* = 0.95, respectively) (Table 1).

### 3.2. Outcomes

There was no significant difference in BBPS scores, BS scores, and withdrawal time between the two groups (*P* = 0.11, *P* = 0.71, and *P* = 0.96, respectively). Moreover, there were no significant differences in AADR, PDR, and adenocarcinoma between the two groups (*P* = 1, *P* = 0.32, and *P* = 1, respectively). Indeed, there were no adverse events, such as bleeding and perforation in both groups (*P* = 1). However, CIT was significantly shorter in the abdominal bandage group compared with the conventional group [279.00 (234.50–305.75) vs. 421.00 (327.00–485.00), *P* < 0.001]. Besides, the CCR and ADR were significantly higher in the abdominal bandage group than in the conventional group (96.7% vs. 88.2%, *P* = 0.01 and 47.5% vs. 32.8%, *P* < 0.001, respectively) (Table 2).

### 3.3. Factors Associated with CCR

**Figure 3:**
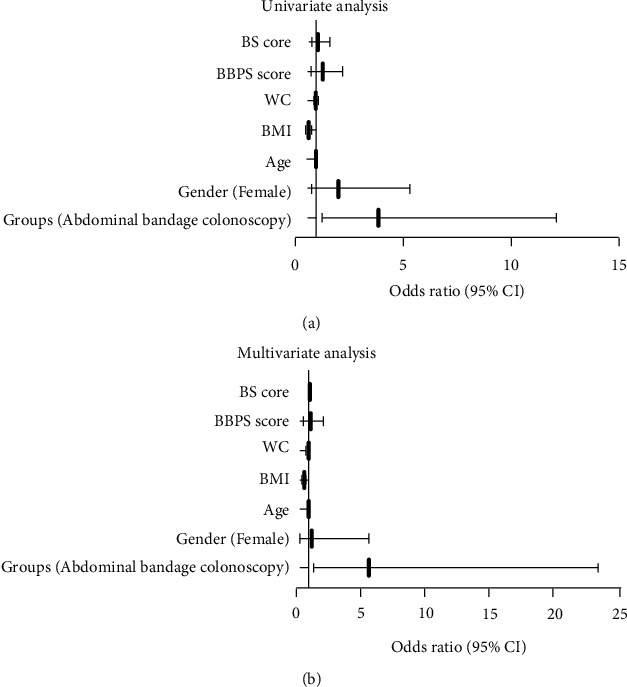
Logistic regression analysis of associated factors with CCR. (a, b) Univariate and multivariate regression analyses showed BMI was a risk factor for lower CCR, whereas the use of abdominal bandages was associated with higher CCR.

Univariate regression analysis revealed that higher BMI was a risk factor for lower CCR [odds ratio (OR) = 0.64; 95% confidence interval (CI) 0.54–0.76; *P* < 0.001], whereas the use of abdominal compression bandage was a useful ancillary maneuver to enhance CCR (OR = 3.87; 95% CI, 1.23–12.12; *P* = 0.02). Other variables (such as gender, age, BBPS score, BS score, and WC) were not associated with CCR (Table 3 and Figure 3(a)). Indeed, multivariate regression analysis showed that BMI was an independent risk predictor of CCR (OR = 0.61; 95% CI, 0.50–0.75; *P* < 0.001), while abdominal compression bandage was an independent protective factor of CCR (OR = 5.63; 95% CI, 1.36–23.33; *P* = 0.02) (Table 3 and Figure 3(b)).

## 4. Discussion

Obesity represents one of the most challenging public health concerns due to its epidemic proportions worldwide and the associated morbidity and mortality [20]. Epidemiological evidence suggests that one-third of the world's population can be classified as overweight or obese, which is an estimated number of 2.1 billion people [21]. In the USA, the prevalence of overweight or obesity is approximately 35% in males and 40.4% in females, which may increase to 86% in adults by 2030 [22, 23]. Furthermore, it has been shown that obesity is one of the most significant predisposing factors for numerous cancers and chronic diseases, such as CRC, hypertension, and diabetes [23, 24]. Moreover, obesity is significantly associated with colorectal adenomas and CRC [2, 4]. Indeed, some studies have reported that childhood obesity tends to increase the incidence of CRC in younger adults [25, 26]. Therefore, more attention should be paid to obesity to prevent CRC.

Several studies have demonstrated that colonoscopy screening can decrease the incidence of CRC by 60% to 90% [27–29]. A complete colonoscopy is crucial for colonoscopy screening. However, obesity tends to decrease the complete colonoscopy rate (CCR) [15]. The main reason may be that it is challenging to minimize looping in an obese abdomen [15]. Therefore, how to improve CCR cannot be ignored. In the present study, we found that an abdominal compression bandage not only shortened the CIT but also improved the CCR compared with conventional colonoscopy. Interestingly, the ADR was significantly higher in the abdominal bandage group compared with the conventional group.

Prechel and Hucke [17] have reported that the hand technique assisting abdominal compression can shorten the CIT and decrease the patients' abdominal pain during routine colonoscopy [17]. Toros et al. [18] have also shown that an abdominal corset providing abdominal compression can decrease the CIT and minimize abdominal pain during routine colonoscopy [18]. In the present study, we found that an abdominal bandage enabling abdomen compression in obese patients significantly shortened the CIT compared with conventional colonoscopy, which was consistent with the above-mentioned studies [17, 18]. However, we did not question patients about their tolerance to colonoscopy because all colonoscopies in this study were performed under sedation. Unfortunately, no study has compared the ADR between two groups of patients with or without abdominal bandages during colonoscopy. Meanwhile, ADR is a key measure of colonoscopy screening quality [30]. In the present study, we showed that the use of abdominal bandages could effectively improve the ADR during colonoscopy. Nevertheless, the specific underlying mechanism remains unclear. It may be important to keep the colonoscope straight in the transverse, sigmoid descending, and sigmoid colon, which possibly improves the bowel visibility and ADR in the abdominal bandage group. It has been reported that a straight colonoscope without a loop can improve the CCR and decrease the cecal insertion time [31]. Moreover, a meta-analysis found that a straight colonoscope without a loop could improve ADR by 16% [32].

Moon et al. [33] have shown that higher BMI is positively associated with prolonged CIT [33]. However, Heieh et al. [34] have reported that WC performs better than BMI in predicting a longer CIT [34]. In the present study, we found that a higher BMI and deprecation of abdominal bandages were associated with lower CCR by univariate and multivariate regression analyses. Interestingly, the ADR was prominently higher in this study compared with our previous study [19]. The main reason might be that different population groups were enrolled in these two studies. For example, obese patients were eligible in this study, whereas the general population was included in the previous study [19].

The strength of this study is that we confirmed that the use of abdominal bandages enabling abdominal compression in obese patients effectively shortened CIT and substantially improved CCR and ADR. However, our study has some limitations. First, the present study was performed in a single center. Second, it was difficult to blind the endoscopists to group allocation. Third, although all endoscopists were experienced, the effect of interobserver heterogeneity could not be ignored. Fourth, position change in both groups was allowed, which might affect the CCR and CIT in both groups. Fifth, the tightness of the bandage was not consistent with all patients in the abdominal bandage group, as this was dependent on the patient's comfort. Sixth, several factors were associated with ADR, such as high-quality bowel preparation, withdrawal time, and endoscopists' skill, among others [30, 34, 35]. However, high-quality bowel preparation (BBPS ≥ 6) and adequate withdrawal time (>360 s) were strictly complied with.

In conclusion, abdominal bandages could shorten CIT and improve CCR and ADR for obese subjects during a routine colonoscopy. However, a multicenter randomized controlled study is urgently required to warrant the feasibility and safety of abdominal bandages for obese subjects undergoing colonoscopy.

## Figures and Tables

**Table 1:** Baseline characteristics.

	Abdominal bandage group	Conventional group	*p* value
Gender, *n* (%)			
Male	82(68.3%)	86(72.3%)	0.51^*a*^
Female	38(31.7%)	33 (27.7%)	

Age (years)	49.53 ± 12.91	48.83 ± 12.40	0.67^*b*^

BMI (kg/m^2^)	27.78 (26.90–29.34)	28.09 (26.99–29.72)	0.32^*c*^

WC (cm)	115 (108–120)	116 (108–118)	0.40^*c*^

Indications for colonoscopy, *n* (%)			
Abdominal pain	23 (19.2%)	26 (21.8%)	0.99^*a*^
Abdominal distension	22 (18.3%)	21 (17.6%)	
Diarrhea	13 (10.8%)	14 (11.8%)	
High CEA	7 (5.8%)	8 (6.7%)	
Screening CRC	38 (31.7%)	35 (29.4%)	

Previous medical history, *n* (%)			
IBS	5 (4.2%)	7 (5.9%)	0.95^*d*^
Hypertension	9 (7.5%)	8 (6.7%)	
Diabetes	4 (3.3%)	5 (4.2%)	
Hypertension and diabetes	3 (2.5%)	4 (3.4%)	
None	99 (82.5%)	95 (79.8%)	

*Note.* BMI, body mass index; WC, waist circumference; CEA, carcinoembryonic antigen; CRC, colorectal cancer; IBS, inflammatory bowel syndrome; ^*a*^Pearson's *χ*^2^ test; ^*b*^Student's *t*-test; ^*c*^Mann–Whitney test; ^*d*^Fisher's exact test.

**Table 2:** Primary outcomes and secondary outcomes.

	Abdominal bandage group	Conventional group	*p* value
BBPS score	6.38 ± 0.94	6.14 ± 0.95	0.11^*a*^
BS score	8.51 ± 0.98	8.48 ± 0.99	0.71^*a*^
Withdraw time (s)	421.00 (369.00–548.00)	418.00 (370.00–550.00)	0.96^*b*^
Adverse events, *n* (%)	0	0	1^*c*^
CIT (s)	279.00 (234.50–305.75)	421.00 (327.00–485.00)	<0.001^*b*^
CCR, *n* (%)	116 (96.7%)	105 (88.2%)	0.01^*d*^

Diagnosis, *n* (%)			
Normal	42 (35.0%)	51(42.9%)	0.65^*e*^
Adenoma	57 (47.5%)	39 (32.8%)	<0.001^*e*^
Advanced adenoma	2 (1.7%)	2 (1.7%)	1^*e*^
Adenocarcinoma	1 (0.8%)	1 (0.8%)	1^*e*^
Polyps	9 (7.5%)	17 (14.3%)	0.32^*e*^
IBD	2 (1.7%)	1(0.8%)	1^*e*^
Others	7 (5.8%)	8 (6.7%)	0.99^*e*^

*Note.* BBPS, Boston Bowel Preparation Scale; BS, Bubble Scale; CIT, cecal insertion time; CCR, complete colonoscopy rate; IBD, inflammatory bowel disease; ^*a*^Student's *t*-test; ^*b*^Mann–Whitney test; ^*c*^Fisher's exact test; ^*d*^Pearson's *χ*^2^ test; ^*e*^Bonferroni method *χ*^2^ test.

**Table 3:** Logistic regression analysis associated the factors with CCR.

	Univariate analysis			Multivariate analysis		
	Crude OR	95% CI	*P* value	Adjusted OR	95% CI	*P* value
Group						
Conventional group	Reference			Reference		
Abdominal bandage group	3.87	1.23–12.12	0.02	5.63	1.36–23.33	0.02

Gender						
Male	Reference			Reference		
Female	2.01	0.76–5.32	0.16	1.23	0.27–5.62	0.79

Age	0.99	0.95–1.03	0.5	0.98	0.93–1.03	0.5
BMI	0.64	0.54–0.76	<0.001	0.61	0.50–0.75	<0.001
WC	0.97	0.90–1.05	0.48	0.98	0.80–1.03	0.12
BBPS score	1.29	0.76–2.19	0.34	1.11	0.58–2.15	0.75
BS score	1.05	0.66–1.68	0.83	1.04	0.55–1.95	0.91

*Note.* CCR, complete colonoscopy rate; BMI, body mass index; WC, waist circumference; BBPS, Boston Bowel Preparation Scale; BS, Bubble Scale; OR, odds ratio; and CI, confidence interval.

## Data Availability

The data are available from the corresponding author.

## Abbreviations

CCR: Complete colonoscopy rate
CIT: Cecal insertion time
AADR: Advanced adenoma detection rate
PDR: Polyp detection rate
BBPS: Boston bowel preparation scale
BS: Bubble scale
CRC: Colorectal cancer
WC: Waist circumference
BMI: Body mass index
FAP: Familial adenomatous polyposis
IBD: Inflammatory bowel disease
SIM: Simethicone
SD: Standard deviation
IQR: Interquartile range
OR: Odds ratio
CI: Confidence interval.
